# Antiprotozoal activity of different *Xenorhabdus* and *Photorhabdus* bacterial secondary metabolites and identification of bioactive compounds using the easyPACId approach

**DOI:** 10.1038/s41598-022-13722-z

**Published:** 2022-06-24

**Authors:** Sebnem Hazal Gulsen, Evren Tileklioglu, Edna Bode, Harun Cimen, Hatice Ertabaklar, Derya Ulug, Sema Ertug, Sebastian L. Wenski, Mustapha Touray, Canan Hazir, Duygu Kaya Bilecenoglu, Ibrahim Yildiz, Helge B. Bode, Selcuk Hazir

**Affiliations:** 1 Department of Biology, Faculty of Arts and Science, Aydin Adnan Menderes University, Aydin, Türkiye; 2 Department of Parasitology, Faculty of Medicine, Aydin Adnan Menderes University, Aydin, Türkiye; 3 Max-Planck-Institute for Terrestrial Microbiology Department, Natural Products in Organismic Interactions, Karl-von-Frisch-Str. 10, 35043 Marburg, Germany; 4 Molekulare Biotechnologie, Fachbereich Biowissenschaften, Goethe Universität Frankfurt, Max-von-Laue-Str. 9, 60438 Frankfurt, Germany; 5 Aydin Health Services Vocational School, Aydin Adnan Menderes University, 09100 Aydin, Türkiye; 6 Faculty of Health Science, Aydin Adnan Menderes University, 09100 Aydin, Türkiye; 7 Senckenberg Gesellschaft für Naturforschung, 60325 Frankfurt, Germany

**Keywords:** Biotechnology, Drug discovery, Microbiology, Molecular biology, Pathogenesis

## Abstract

Natural products have been proven to be important starting points for the development of new drugs. Bacteria in the genera *Photorhabdus* and *Xenorhabdus* produce antimicrobial compounds as secondary metabolites to compete with other organisms. Our study is the first comprehensive study screening the anti-protozoal activity of supernatants containing secondary metabolites produced by 5 *Photorhabdus* and 22 *Xenorhabdus* species against human parasitic protozoa, *Acanthamoeba castellanii, Entamoeba histolytica, Trichomonas vaginalis, Leishmania tropica* and *Trypanosoma cruzi,* and the identification of novel bioactive antiprotozoal compounds using the easyPACId approach (easy Promoter Activated Compound Identification) method. Though not in all species, both bacterial genera produce antiprotozoal compounds effective on human pathogenic protozoa. The promoter exchange mutants revealed that antiprotozoal bioactive compounds produced by *Xenorhabdus* bacteria were fabclavines, xenocoumacins, xenorhabdins and PAX peptides. Among the bacteria assessed, only *P. namnaoensis* appears to have acquired amoebicidal property which is effective on *E. histolytica* trophozoites. These discovered antiprotozoal compounds might serve as starting points for the development of alternative and novel pharmaceutical agents against human parasitic protozoa in the future.

## Introduction

Infectious diseases are caused by the invasion and continued presence of pathogenic microorganisms such as viruses, bacteria, fungi, protozoa, nematodes, etc. in a host`s body organ, tissue, or cells. Protozoa in particular, such as *Acanthamoeba castellanii* (*A. castellanii), Entamoeba histolytica* (*E. histolytica*)*, Trichomonas vaginalis* (*T. vaginalis*)*, Leishmania tropica* (*L. tropica*) and *Trypanosoma* spp*.*, are eukaryotic single-celled organisms that are the leading cause of numerous untold deaths and devastating chronic diseases worldwide, especially in underdeveloped and developing countries of sub-Saharan Africa, Asia and South America^1–3^. They are transmitted directly or indirectly through contact, air contaminated food or water, or by vectors from infected humans and animals to healthy others^1^. Poverty, inadequate sanitation and unhygienic living conditions, malnutrition, suitable climatic factors, ineffective anti-parasitic drugs, inept vector control interventions, insecticide resistance are some of the factors that contribute to the persistence and incidence of such parasitic infectious diseases in various parts of the world^4–6^.

Since protozoan parasites are eukaryotic organisms that share functional homology with mammalian cells, currently available drugs for the treatments of parasitic diseases are generally toxic to human cells and have adverse side effects^7,8^. Owing to these undesired effects and considering the development of resistant strains of parasites against pharmaceutical products, new drugs with different modes of action on target parasites and minimal toxicity to host cells are urgently required^9,10^.

Natural products (or secondary metabolites) have been proven to be an important starting point for the development of new drugs. Screening natural products provide the chance of discovering new molecules with unique structure, high activity, and selectivity^11^. The most important natural product sources in nature are fungi^12,13^, plants^14^ and bacteria^11,15,16^. Various fungi and bacteria produce antimicrobial compounds as secondary metabolites to compete with other organisms. One of the sources of novel bioactive therapeutics against parasites are insect pathogenic *Photorhabdus* and *Xenorhabdus* bacteria. These bacteria encode several putative biosynthetic pathways for natural product biosynthesis^17–19^ of which several of them are conserved since they fulfill important ecological functions in their ecological niche^20^. *Photorhabdus* and *Xenorhabdus* bacteria are associated with entomopathogenic nematodes which are obligate and lethal insect parasitic organisms^21,22^. When these nematodes penetrate an insect host, they release their mutualistic bacteria into the insect hemolymph and within 48 h the insect host is killed because of bacterial toxins and enzymes^23,24^. Furthermore, to protect the nematode-infected cadaver from opportunistic microorganisms (e.g., bacteria, fungi, protozoa, and viruses) both *Xenorhabdus* and *Photorhabdus* bacteria produce a variety of natural products that have antimicrobial activities^19,25,26^. Although several studies have reported the antibacterial^27–30^, antifungal^29–35^, and insecticidal^36–38^ activities, only very few studies have investigated the antiprotozoal effect of the secondary metabolites produced by these bacteria^39,40^. Currently, more than 40 different species of *Photorhabdus* and *Xenorhabdus* bacteria have been identified^23,41^ that produce different sets of natural products^42^. The aim of our study was to investigate natural products produced by five *Photorhabdus* and 22 *Xenorhabdus* species against human parasitic protozoa, *A. castellanii, E. histolytica, T. vaginalis, L. tropica,* and *Trypanosoma cruzi* (*T. cruzi*)*,* and the identification of novel bioactive antiprotozoal compounds by using the easyPACId (easy Promoter Activated Compound Identification) approach^43^.

## Material and methods

### Bacterial sources and preparation of cell-free supernatants

The cell-free supernatants of 22 *Xenorhabdus* and 5 *Photorhabdus* species were tested against human parasitic protozoa (Table 1). All bacteria strains were obtained from the Bode lab and were kept at − 80 °C as stock culture until use.

**Table 1:** Bacterial species used in antiprotozoal activity tests.

	Bacteria species
1	*Xenorhabdus nematophila* ATCC 19061
2	*X. bovienii* SS-2004
3	*X. vietnamensis* DSM 22392
4	*X. cabanillasii* JM26-1
5	*X. szentirmaii* DSM 16338
6	*X. stockiae* DSM 17904
7	*X. ehlersii* DSM 16337
8	*X. koppenhoferi* DSM 18168
9	*X. indica* DSM 17382
10	*X. maulenoii* DSM 17908
11	*X. poinarii* G6
12	*X. griffiniae* DSM 17911
13	*X. ishibashi* DSM 22670
14	*X. doucetiae* DSM 17909
15	*X. innexi* DSM 16336
16	*X. japonica* DSM 16522
17	*X. khoisanae*
18	*X. beddingii* DSM 4764
19	*X. budapestensis* DSM 16342
20	*X. miraniensis* DSM 17902
21	*X. hominickii* DSM 179903
22	*X. kozodoii* DSM 17907
23	*Photorhabdus kayaii* DSM 15194
24	*P. namnaoensis* PB 45.5
25	*P. laumondii* TTO1
26	*P. akhurstii* DSM 15138
27	*P. thracensis* DSM 15199

A loopfull of bacteria taken from stock culture was inoculated to Luria Bertani (LB) (Merck) agar medium and incubated at 30 °C for 24 h. A single colony was picked and inoculated to 10 ml sterilized Tryptic Soy Broth (TSB) medium (Merck) and cultivated at 30 °C for 24 h to be used as overnight culture. Subsequently, 1 ml from overnight culture was transferred to 50 ml sterilized TSB medium and incubated at 30 °C and 150 rpm for 120 h (it is known that these bacteria produce the most secondary metabolite after 120 h)^30,44^. To obtain cell-free supernatant, the bacterial broth was centrifuged at 10,000 rpm for 10 min at 4 °C. The supernatant was collected carefully and filtered through a 0.22 μm Millipore filter (ISOLAB)^45^. An aliquot of the filtrated suspension was streaked onto NBTA agar to verify the absence of bacterial cells^46^. The supernatants were poured into the 50 ml sterile centrifuge tubes (Corning, NY) and kept at − 20 °C for up to 2 weeks prior to use^47,48^.

### In vitro cultures of parasitic protozoons

Axenic cultures of *A. castellanii* trophozoites (ATCC 30010) were maintained in liquid PYG (protease peptone—yeast extract—glucose) medium supplemented with penicillin G (500 U/ml) and streptomycin (50 μg/ml)^49^ (Pérez-Serrano et al. 2000). The cultures were refreshed weekly in 25 ml cell culture flasks (Sigma) and incubated at 30 °C, until use^50,51^. Cells from the culture medium were harvested by centrifugation at 2000 rpm for five minutes and washed three times with Phosphate-Buffered Saline (PBS). *Acanthamoeba castellanii* trophozoites adhering to flasks were collected by placing the flasks on ice for 30 min with gentle agitation^52,53^.

*Entamoeba histolytica* (ATCC 30459) strain was kindly provided by Dr. Charles Graham Clark from the London School of Hygiene and Tropical Medicine. *Entamoeba histolytica* trophozoites were cultured axenically in LYI medium (880.0 ml LYI Broth, 20.0 ml Vitamin Mixture, 100.0 ml Heat Inactivated Adult Bovine Serum) supplemented with penicillin G (500 U/ml) and streptomycin (50 μg/ml)^54^. The cultures were routinely maintained by subculturing into screw capped test tube containing 7 mL of LYI medium^55,56^.

*Trichomonas vaginalis* (ATCC 30001) trophozoites were grown in Diamond’s trypticase yeast-extract maltose (TYM) medium (0.5 mg of L-cysteine HCl, 0.1 g of ascorbic acid, 0.4 g of K_2_HPO_4_, 0.4 g of KH_2_PO_4_, 10 g of trypticase, 2.5 g of maltose and 10 g of yeast extract in one ml of distilled water, pH:6) supplemented with 100 IU/ml streptomycin, 100 IU/ml penicillin and 10% heat-inactivated Fetal Bovine Serum (FBS). *T. vaginalis* subcultures were cultured regulaly to maintain viability and for use in the assays^57^.

*Leishmania tropica* (ATCC 50129) promastigotes were routinely cultured at 27 °C in RPMI-1640 medium (Sigma) supplemented with 10% heat-inactivated FBS (Cegrogen, Stadtallendorf-Germany). The culture was sustained in 25 ml flasks and stationary phase of promastigotes were obtained^58^.

*Trypanosoma cruzi* (CBU-TC01) trypomastigotes were obtained from the parasite biobank of Manisa Celal Bayar University School of Medicine Department of Parasitology Manisa, Turkey. The trypomastigotes were incubated at 27 °C in RPMI 1640 medium supplemented with 10% heat-inactivated FBS, 200 U penicillin/ml, and 0.2 mg streptomycin/ml. Subcultures were maintained in 25 ml flasks until use in the experiments^59,60^.

### In vitro antiprotozoal activity of bacterial secondary metabolites

Except for *E. histolytica*, the microdilution method was used to assess the antiprotozoal activity of the bacterial supernatants against *A. castellanii*, *T. vaginalis*, *L. tropica* and *T. cruzi*. The four parasites were seeded in 96-well Microtiter plates (Greiner, Germany) and the supernatants were applied at serial concentrations ranging from 10% to 1.25%. Briefly, Trophozoites of *A. castellanii* and *T. vaginalis* were adjusted to 5 × 10^4^ and 2 × 10^6^ cells/mL, respectively. The density of *L. tropica* promastigotes and trypomastigotes of *T. cruzi* were adjusted to 1 × 10^6^ cells/mL^61,62^. Plates with the isolates were incubated at 30 °C for 24 h, 37 °C for 48 h and 27 °C for 72 h for *A. castellanii*, *T. vaginalis*, *L. tropica,* and *T. cruzi* respectively. Screw capped test tubes were used for *E. histolytic*a instead of the plates used for other parasites. *Entamoeba histolytica* trophozoites (200 µl of 3 × 10^5^ cells/mL) were inoculated into the tubes containing 1.8 ml of fresh axenic LYI medium with the bacterial supernatants at final concentrations of 10%, 5%, 2.5%, and 1.25%. The tubes were incubated at 37 °C for 48 h^56^.

Two methods were used to determine the antiprotozoal effects of the bacterial supernatants in vitro. To assess the anti-leishmanial activity was performed by using the XTT (sodium 3,39- [1- (phenylaminocarbonyl)-3,4-tetrazolium]-bis (4-methoxy-6-nitro) benzene sulfonic acid hydrate) cell proliferation kit (Roche Molecular Biochemicals, Mannheim, Germany) as previously described^63^.

Cell viability assay test was used for *A. castellanii*, *T. vaginalis*, *E. histolytica* and *T. cruzi.* The assay was evaluated by adding 0.1% trypan blue stain (TB) [the number of live (unstained) and dead (stained)] using a hemocytometer^64,65^. The parasite mortality (in %) for each bacterial supernatants sample was caluclated according to the formula: % Mortality of parasites = (Control Negative-Test sample) × 100%/Control negative. Only 100% inhibition of the parasite was considered when no motile parasite was observed.

Two negative and one positive control group were included in each experiment. Bacterial culture medium (TSB) and parasite medium was used as a negative control. Metronidazole (Specia Rhone Poulenc Rorer, Paris, France) for *T. vaginalis* and *E. histolytica*, Chlorhexidine (Sigma, Spain) for *A. castellanii*, N-methyl meglumine (Glucantime™, Rhone Poulenc, France) for *L. tropica* and Benzimidazole (Sigma, Spain) for *T. cruzi* were used as positive controls. Each assay was performed at least three times in triplicate.

### Identification of antiprotozoal compounds using the easyPACId method

#### Generating promoter exchange mutant strains

The easyPACId approach method recently developed by Bode et al.^43^ was used to identify the antiprotozoal compounds in *Xenorhabdus* spp. bacteria. Briefly, ∆*hfq* mutants of each bacterial species (*X. budapestensis, X. cabanillasii, X. doucetiae, X. hominickii, X. nematophila, X. stockiae* and *X. szentirmaii*) were first generated and then the native promoter regions of selected natural product biosynthetic gene clusters of these bacteria were exchanged with the chemically inducible promoter P_BAD_ (addition of L-arabinose) via integration of the pCEP-KM plasmid^43,66^. This allows the selective production of a desired single natural product compound class and enables direct bioactivity analysis of the corresponding supernatant instead of time-consuming isolation of single compound(s) from analytically complex wild type extracts. The generation of the described *Xenorhabdus spp.* ∆*hfq* as well as *Xenorhabdus spp.* ∆*hfq* pCEP-KM-xy mutants listed in Table 3 (xy describes the locus of the first biosynthetic gene cluster) is described in detail by Bode et al.^43^.

### Obtaining cell-free supernatants from different *Xenorhabdus spp.* ∆*hfq* promoter exchange mutants

A single *Xenorhabdus spp.* ∆*hfq* pCEP-KM-xy mutant colony, cultivated on LB agar supplemented with a 50 μg/mL final concentration of kanamycin at 30 °C for 48 h, was transferred into LB medium (10 mL) also supplemented with a 50 μg/mL final concentration of kanamycin and incubated at 150 rpm and 30 °C. Then, this overnight culture was inoculated into a fresh 20 mL LB with the final optical density (OD_600_) adjusted to 0.1. After an hour incubation at 30 °C, these cultures were induced with 0.2% L-arabinose and incubated again for 72 h at 150 rpm and 30 °C^43,67^. Cultures of non-induced mutants contained no L-arabinose. The cell-free supernatants were obtained by centrifugation at 10.000 rpm for 20 min in 50 ml Falcon tubes at 4 °C and filteration through a 0.22-µm Millipore filter (Thermo scientific, NY) to ensure total removal of bacterial cells^34^. The cell free supernatants were stored at − 20 °C and used within 2 weeks^48^.

### Testing the antiprotozoal activity of cell-free supernatants of mutant strains

Antiprotozoal activity of 5-day-old cell-free supernatants of wild type strains, as well as induced (with arabinose) and non-induced (without arabinose) promoter exchange mutant strains were tested in microdilution bioassay as previously described in the in vitro antiprotozoal activity tests section.

### Anti-protozoal activity of bioactive extracts obtained from hfq mutants

As a last step, extracts containing identified anti-protozoal compounds were tested again on the parasite species at different concentrations ranged from 10 to 0.078% (v/v). The same experimental method used in antiprotozoal activity tests was carried out here.

Anti-protozoal bioactive compound extraction was performed by culturing induced *X. nematophila* Δ*hfq*_pCEP_ kan_XNC1_1711 for xenocoumacin production and *X. doucetiae* Δ*hfq*_P_BAD__ PAX_km for PAX peptide production in LB (6L) with 2% XAD® resin at 30 °C for 3 days. Afterwards the resin was exhaustively extracted with methanol (3 × 2 L) at 24 ± 1 °C and concentrated under reduced pressure to give a crude extract enriched by the desired natural compound class. The extracts were then dissolved in DMSO and prepared as a stock solution with distilled water. Fabclavine was obtained by concentrating the supernatant of the induced *X. cabanillasii* Δ*hfq*_128-129 culture 10-fold using an evaporator.

### Statistical analysis

Differences in antiprotozoal activity of the supernatants were compared with one-way ANOVA and the means separated using Tukey’s test. *P* values < 0.05 were considered as significant^68^. The results are reported as mean ± SD for all values.

### Ethics approval

This article does not contain any studies with human participants or animals performed by any of the authors.

## Results

### In vitro antiprotozoal activity tests

#### Acanthamoeba castellanii

The in vitro activity assays against the trophozoite of *A. castellanii* showed that 12 of 22 *Xenorhabdus* species exhibited effective antiprotozoal activity whereas, none *Photorhabdus* strain showed any activity (Table 2). At 10% supernatant concentration, *A. castellanii* cell mortality ranged between 78 and 100% depending on *Xenorhabdus* species. Relative to the negative control, 12 of 22 tested *Xenorhabdus* supernatants caused significant *A. castellanii* mortality (F = 1828.80; df = 28, 232; *P* < 0.0001). Chlorhexidine used as positive control showed 100% mortality and no statistical difference was observed between chlorhexidine and *X. budapestensis, X. cabanillasii, X. doucetiae* and *X. innexii* supernatants*.* At 5% concentration of bacterial supernatants, the highest level of mortality (> 95%) was exhibited by *X. budapestensis, X. cabanillasii, X. innexii* and chlorhexidine (which were not statistically different from each other). All *Xenorhabdus* species with antiprotozoal activity presented statistically significant mortality compared to the negative controls (F = 1357.38; df = 28, 232; *P* < 0.0001). In the following concentration (2.5%), the supernatants of *X. budapestensis, X. cabanillasii* and *X. innexii* exhibited more than 90% mortality on *A*. *castellanii* trophozoites and no significant difference was observed between this group and chlorhexidine (Table 2). *Xenorhabdus miraniensis* and *X. nematophila* supernatants caused the lowest mortality (55%) on the trophozoites. Despite this, there was a significant difference between all effective 12 *Xenorhabdus* supernatant treatments and negative controls (F = 653.63; df = 28, 232; *P* < 0.0001). At the lowest concentration of tested bacterial supernatants (1.25%), *X. budapestensis* and *X. innexii* species showed equal mortality with chlorhexidine. Following these species, supernatants of *X. cabanillasii*, *X. doucetiae*, *X. hominickii, X. stockiae,* and *X. szentirmaii* were more effective compared with the other treatments. Even at highly diluted concentrations of the supernatants compared to negative controls, significant mortalities were obtained (F = 550.64; df = 28, 232; *P* < 0.0001) (Table 2).

**Table 2:**
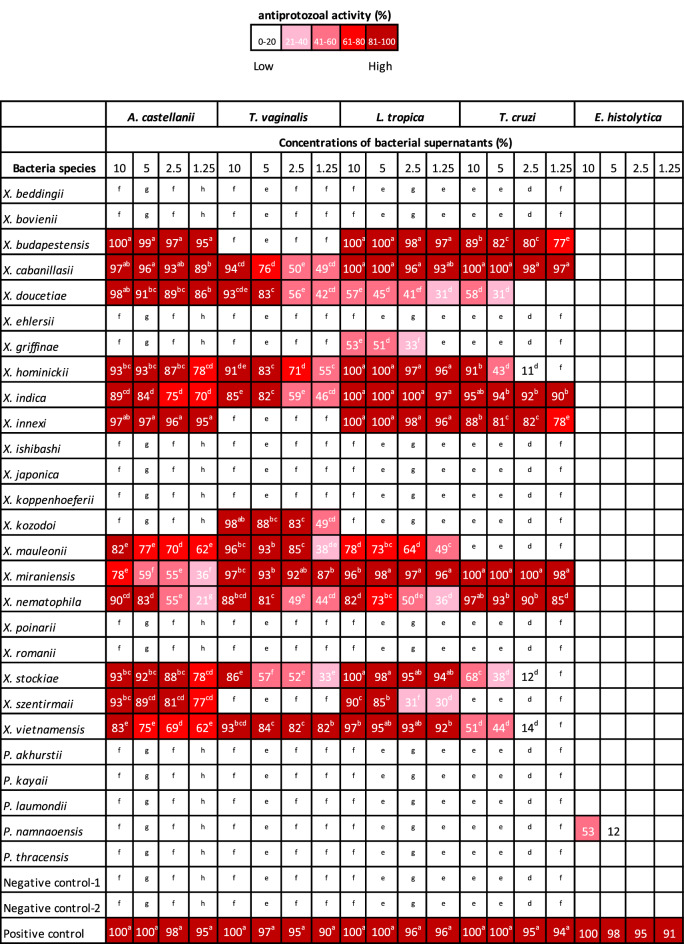
Antiprotozoal activity of cell-free culture supernatants of *Xenorhabdus* and *Photorhabdus* spp. against different human parasitic protozoa species.

Bioactivities are shown for none (white) to highest activity (dark red) in the different assays. Negative control-1: Bacterial culture medium (TSB), Negative control-2: Parasite growth medium. Positive controls: Metronidazole for T*. vaginalis* and E. histolytica, Chlorhexidine for *A. castellanii*, N-methyl meglumine for *L. tropica* and Benzimidazole for *T. cruzi*. Columns with a common superscript letter do not differ significantly at *P* = 0.05).

#### Trichomonas vaginalis

It was noted that 10 of the 22 tested *Xenorhabdus* supernatants were significantly lethal against *T. vaginalis* when compared with negative controls. Of these, *X. cabanillasii, X. doucetiae, X. hominickii, X. kozodoii, X. mauleonii, X. miraniensis* and *X. vietnamensis* species displayed more than 90% mortality against *T. vaginalis* at 10% supernatant concentration (F = 334.60; df = 29, 240; *P* < 0.0001) (Table 2)*.* At 5% concentration, though the positive control metronidazole caused statistically significant mortality compared to the supernatant treatments, all the other effective bacterial supernatants exhibited mortality ranging between 57 and 93% (F = 288.74; df = 29, 240; *P* < 0.0001). Among the effective bacterial supernatants, *X. miraniensis, X. mauleonii, X. kozodoii* and caused 92, 85 and 83% *T. vaginalis* mortality, respectively, at 2.5% concentration (F = 288.74; df = 29, 240; *P* < 0.0001). There was a significant difference between the effective treatments and controls (F = 237.78; df = 29, 240; *P* < 0.0001). *Xenorhabdus miraniensis* and *X. vietnamensis* still exhibited more than 80% mortality, when the supernatants were diluted to the lowest concentration (1.25%) (F = 255.82; df = 29, 240; *P* < 0.0001). However, none of the *Photorhabdus* species caused *T. vaginalis* mortality at any concentration (Table 2).

#### Leishmania tropica

*Xenorhabdus budapestensis, X. cabanillasii, X. hominickii, X. indica, X. innexii* and *X. stockiae* supernatants caused 100% mortality at the highest tested concentration (10%) against the promastigote form of *L. tropica*. No differences occurred between this treatment group and positive control (*P* > 0.05). Following those bacteria species *X. vietnamensis, X. miraniensis,* and *X. szentirmaii* supernatants exhibited 97, 96 and 90% mortality, respectively. The other effective bacterial supernatants presented mortalities that ranged between 53 and 82%. There was a significant difference between all effective treatment groups and negative control (F = 880,33; df = 29, 240; *P* < 0.0001) (Table 2).

Similarly, at 5% concentration, *X. budapestensis, X. cabanillasii, X. hominickii, X. indica, X. innexii, X. miraniensis, X. stockiae* and Stibogluconate caused 98–100% *Leishmania* mortalities. No significant difference was observed among these groups. Although *X. doucetiae* presented the least *Leishmania* mortality (40%), there were statistically significant differences between all effective treatments and negative controls (F = 232.16; df = 29, 240; *P* < 0.0001) (Table 2). At 2.5% supernatant concentration, *X. budapestensis, X. cabanillasii, X. hominickii, X. indica, X. innexii, X. miraniensis, X. stockiae* and *X. vietnamensis* showed the highest efficacy (93–100%), whereas *X. griffinae* and *X. szentirmaii* supernatants exhibited the lowest mortalities (33 and 31%, respectively). However, there was a significant difference between all effective treatments and negative control groups (F = 425.10; df = 29, 240; *P* < 0.0001) (Table 2).

Bacterial supernatant of *X. budapestensis, X. cabanillasii, X. hominickii, X. indica, X. innexii, X. miraniensis* and *X. stockiae* were still as effective as N-methyl meglumine even at 1.25% concentration. There was a significant difference between the effective bacterial supernatants and negative controls (F = 393.67; df = 29, 240; *P* < 0.0001) (Table 2).

#### Trypanosoma cruzi

At 10% concentration, 10 of 27 bacterial supernatants showed antiprotozoal activity against *T. cruzi* trypomastigotes. Among the bacterial species, *X. cabanillasii* and *X. miraniensis* exhibited 100% mortality followed by *X. nematophila, X. indica, X. hominickii, X. budapestensis* and *X. innexi* supernatants (88–97%). There was a statistically significant difference between effective bacterial supernatants and negative controls (F = 288.53; df = 29, 240; *P* < 0.0001) (Table 2).

When the bacterial supernatants were diluted to 5% concentration, *X. cabanillasii* and *X. miraniensis* still exhibited 100% mortality. *Xenorhabdus indica, X. nematophila, X. budapestensis* and *X. innexi* supernatans displayed between 94 and 81% mortality. The effect of *X. hominickii* dropped drastically from 91 to 43%. Significant differences were observed between negative controls and the effective supernatants (F = 178.60; df = 29, 240; *P* < 0.0001). At 2.5% concentration of the supernatants, *X. miraniensis* and *X. cabanillasii* had the highest mortality (98–100%) and there was no significant difference between this bacterial species and Benzimidazole (positive control). Among the other bacterial supernatans, *X. budapestensis, X. indica, X. innexi* and *X. nematophila* also caused relatively high mortality which ranged between 80 and 92%. However, *X. doucetiae, X. hominicki, X. stockiae,* and *X. vietnamiensis* lost their antiprotozoal effects at this concentration. A significant statistical difference was observed between the six effective bacterial supernatants and negative controls (F = 589.60; df = 29, 240; *P* < 0.0001) (Table 2).

At the lowest tested concentration (1.25%), *X. cabanillasii* and *X. miraniensis* maintained their high activity (97 and 98% mortality, respectively). *Xenorhabdus indica* followed this group with a mortality of 90%. There was a statistical difference between negative control groups and six bacterial supernatants (F = 929.48; df = 29, 240; *P* < 0.0001) (Table 2).

#### Entamoeba histolytica

Unlike *A. castellanii, E. histolytica,* a different amebic parasite*,* was resistant to the secondary metabolites of *Xenorhabdus* and *Photorhabdus* bacteria. Only *P. namnaoensis* species showed significant mortality compared to the negative controls (F = 1.02; df = 28,232; *P* < 0.0001) (Table 2). Positive control (metronidazole) and *P. namnoensis* supernatant caused 100% and 53% cell mortality on *E. histolytica* trophozoites, respectively. However, the other 26 of 27 tested species exhibited only between 0 and 6% mortality at 10% concentration.

### Identification of antiprotozoal compounds

**Figure 1:**
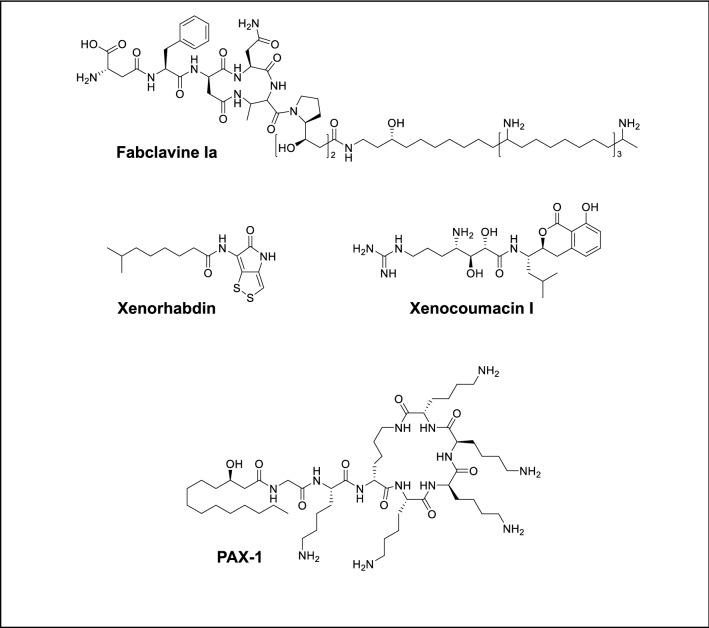
Chemical structure of antiprotozoal natural products from *Xenorhabdus* species.

The promoter exchange mutants in Δ*hfq* background revealed that antiprotozoal bioactive compounds produced by *Xenorhabdus* bacteria were fabclavines, xenocoumacins, xenorhabdins and PAX peptides (Table 3, Fig. 1). The supernatants obtained from induced mutants showed very high mortality against the parasite cells, non-induced mutants of the same compounds exhibited no activity (Table 3).

**Table 3:**
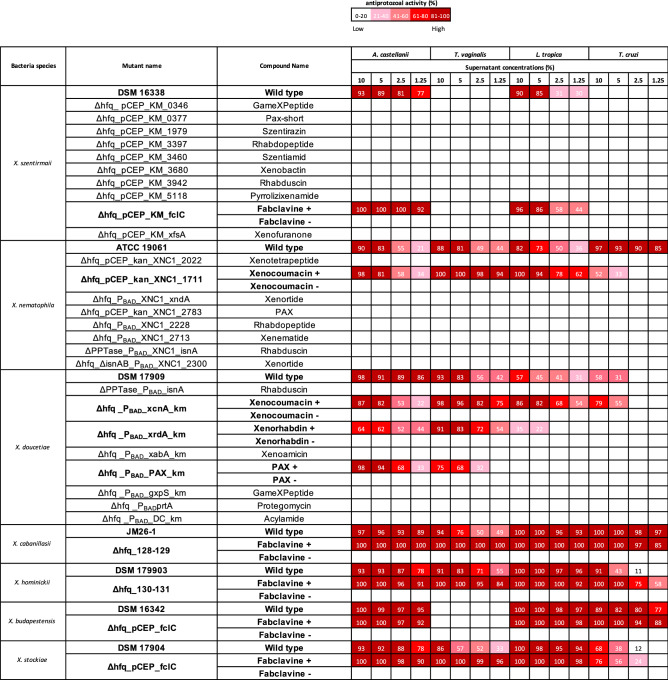
Activity of promoter exchange mutants and their respective natural products against the *Acanthamoeba castellanii, Trichomonas vaginalis, Leishmania tropica* and *Trypanasoma cruzi.*

Activity of all easyPACId strains (delta hfq mutants) was determined after induction of the PBAD Promoter with L-arabinose. Bioactivities are shown for none (white) to highest activity (dark red) in the different assays. In order to confirm the active compounds, these are also shown non-induced (−) and induced (+).

Fabclavines produced by *X. cabanillasii, X. hominickii* and *X. stockiae* species had antiprotozal activity against *A. castellanaii, T. vaginalis L. tropica* and *T. cruzi* parasites. Fabclavines produced by *X. budapestensis* was not effective against *T. vaginalis. Xenorhabdus szentirmaii* also produces fabclavines being only effective against *A. castellanii* and *L. tropica* with no antiprotozoal activity against *T. vaginalis* and *T. cruzi*.

Xenocoumacins produced by *X. nematophila* species was the bioactive antiprotozoal compound against all tested pathogens. In contrast to other species, *X. doucetiae* species produce more than one antiprotozoal compound. Δhfq_P_BAD__PAX_km of *X. doucetiae* producing PAX peptides exhibited antiprotozoal effect on *A. castellanii* and *T. vaginalis*, but *L. tropica* was killed by xenocoumacins and xenorhabdins. *Xenorhabdus doucetiae* Δhfq_P_BAD__xcnA_km showed antiprotozoal activity only with xenocoumacins against *T. cruzi* (Table 3). The active compound in *P. namnaoensis* which was the only species that caused mortality on *E. histolytica* trophozoites was not identified due to the lack of promoter exchange mutants of this species.

### Anti-protozoal activity of bioactive extracts obtained from hfq mutants

**Figure 2:**
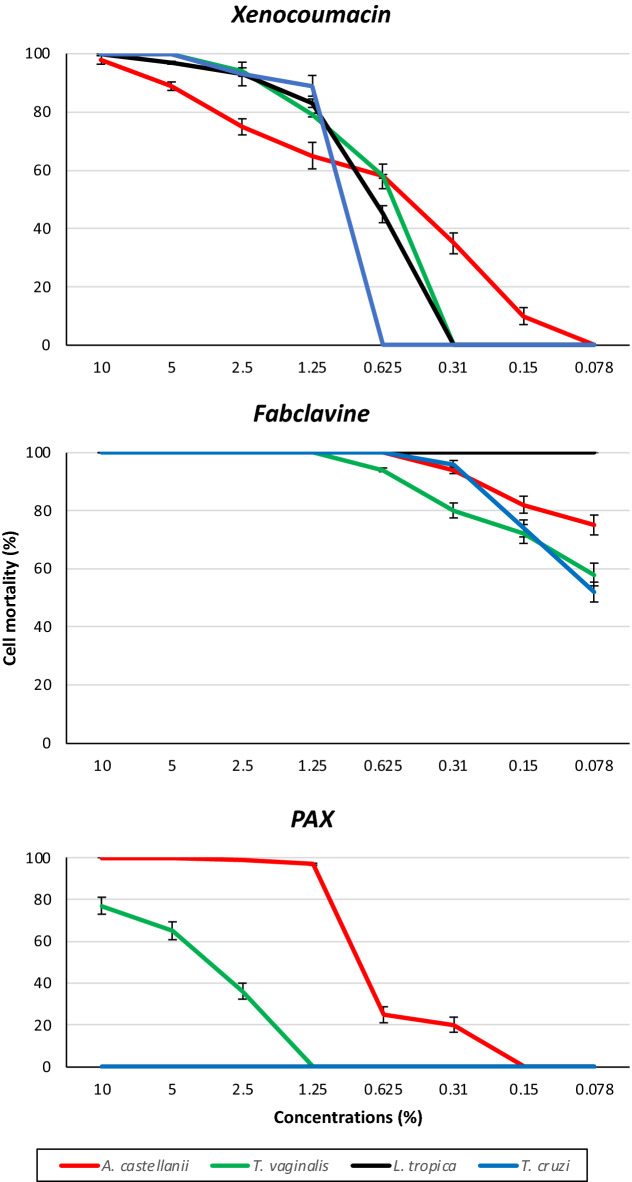
Antiprotozoal activity of supernatants containing bioactive compounds. Xenocoumacins, fabclavines and PAX peptides were obtained from *Xenorhabdus nematophila, Xenorhabdus cabanillasii* and *Xenorhabdus doucetiae,* respectively.

Supernatants containing xenocoumacins, fabclavines and PAX peptides showed variable activity depending on parasite species and concentrations; no mortality was observed in the control (Fig. 2). Overall fabclavine molecules were highly effective on all tested parasite species even at very low concentrations.

## Discussion

Our data revealed that *Xenorhabdus* and *Photorhabdus* produce antiprotozoal compounds effective on human pathogenic protozoa. However, not all *Xenorhabdus* or *Photorhabdus* species showed this activity. Except for *E. histolytica*, only some of *Xenorhabdus* species exhibited antiprotozoal activity. It was reported that *Xenorhabdus* bacteria produce broad-spectrum compounds with various activity against several organisms such as bacteria, fungi, insects, nematodes, mites, protozoa etc. to protect and bioconvert the host cadaver^69–72^. With the easyPACId approach we were able to assign the described activities on respective natural products from *Xenorhabdus*.

The bioactivity of fabclavines could be confirmed for *X. budapestensis, X. cabanillasii, X. hominickii, X. stockiae* and *X. szentirmaii* mutants. Biochemically fabclavines are peptide/polyketide hybrids connected to a polyamine moiety generated by a fatty acid/polyketide synthase with similarity to enzymes producing polyunsaturated fatty acids (PUFAs)^67,73,74^. Fabclavines 1a and 1b exhibit various bioactivities against different bacterial, fungal and protozoal organisms^73^ and due to such broad-sprectrum activity, fabclavines might serve as protective agents against saprophytic food competitors/microorganisms that attack insect cadavers; this enables *Xenorhabdus/Steinernema* to maintain a monoculture in the infected insect^73^. Fabclavines are structurally very similar to zeamines identified in *Serratia plymuthica* so might similarly permeabilize artificial bacterial and eucaryotic model membranes^75,76^. A structurally yet-undentified fabclavine derivative from *X. innexi* (Xlt) induces membrane degradation at low concentrations in selected mosquito cell lines which led to apoptosis^77^. Production of fabclavine is widespread in *Xenorhabdus* strains whereas, except for *Photorhabdus asymbiotica,* other *Photorhabdus* species do not produce fabclavines^42^. This can explain partially why none of our tested *Photorhabdus* species showed antiprotozoal activity*.* However, *X. bovienii* is a producer of only the polyamine part of fabclavine^74^ and it did not exhibit any activity. There are 32 different types of fabclavine with important variations among their activity^67^.

*Xenorhabdus nematophila* and *X. doucetiae* species do not produce fabclavine^42^ but they are effective species on tested parasites except for *E. histolytica*. According to promoter exchange data, it became obvious that *X. nematophila* and *X. doucetiae* perform this task with different compounds. Xenocoumacins are produced using a hybrid nonribosomal peptide synthetase (NRPS) multienzyme (XcnA-N) and polyketide synthase (PKS)^78–80^. When tested for biological activity against *T. b. rhodesiense*, *T. cruzi*, *L. donovani* and *Plasmodium falciparum,* good activities were observed against *T. b. rhodesiense* and *P. falciparum*^80^. Possibly, xenocoumacins inhibit protein biosynthesis in these organisms^80,81^. However, xenocoumacins are not widely distributed in *Xenorhabdus* spp. as one would expect. Among 25 *Xenorhabdus* and *Photorhabdus* strains, xenocoumacins or the corresponding biosynthetic gene cluster were only be identified from seven *Xenorhabdus* subspecies (*X. nematophila, X. indica, X. miraniensis, X. stockiae, X. kozodoii, X. mauleonii* and *X. doucetiae*)^42^

According to our data, we have determined that xenorhabdins and PAX peptides produced by *X. doucetiae* are other effective secondary metabolites. Xenorhabdins are dithiolopyrrolone compounds^82^ and it is reported that they have antibacterial, antifungal, and insecticidal activities^83–85^. Their suggested mode of action is the inhibition of RNA synthesis affecting translation as similar to xenocoumacins^86,87^. PAX peptides are lysine-rich cyclolipopeptides. Gualtieri et al.^88^ first described five PAX peptides from *X. nematophila* and then additional eight PAX peptides were identified, and their structures elucidated by Fuchs et al.^73^. Three NRPS genes (*paxABC*) are responsible for the biosynthesis of the PAX compounds. These peptides have antifungal and antibacterial activity. They exhibited strong anti-fungal activity against the opportunistic human pathogen *Fusarium oxysporum* as well as several plant pathogenic fungi^88^.

Interestingly, among the tested 27 *Xenorhabdus* and *Photorhabdus* strains only *P. namnoensis* appears to have acquired amoebicidal property which is effective on *E. histolytica* trophozoites. The bioactive compound responsible for this activity and its mode of action needs to be identified in the future.

The determined bioactive compounds may offer new opportunities for treating important parasitic diseases or be useful as lead compounds in the development of new antiprotozoal agents. For this purpose, new bioactive compounds should have no or very low cytotoxicity on human cells. Bode et al.^43^ tested the efficacy of bioactive compounds isolated from *Xenorhabdus* and *Photorhabdus* bacteria on the human microvascular endothelial cell (EC) line (CDC.EU.HMEC-1). Fabclavine, PAX peptide, xenocoumacin and xenorhabdin had no or low impact on the metabolic activity, apoptosis and cell cycle G2-block. However, xenocoumacin and xenorhabdin exhibited toxic effects on cell proliferation.

In conclusion, this is the first extensive study screening the anti-protozoal activity of *Xenorhabdus* and *Photorhabdus* secondary metabolites against important human parasites *A. castellanii, E. histolytica, T. vaginalis, L. tropica* and *T. cruzi* and using the easyPACId technique to identify new potential antiprotozoal compounds. Future studies should investigate in detail the mode of action of these promising antiprotozoal compounds. Also, after a close structural investigation of these NPs, novel and safer pharmaceutical drugs can be potentially designed and synthesized.

## Data Availability

All data generated from this study are included in this article.

## References

[1] Farrar J, Hotez PJ, Junghanss T, Kang G, Lalloo D, White N (2013). Manson’s Tropical Diseases.

[2] Despommier D, Griffin DO, Gwadz R, Hotez P, Knirsch C (2017). Parasitic Diseases.

[3] World Health Statistics. *Monitoring Health for the SDGs, Sustainable Development Goals*. https://apps.who.int/iris/handle/10665/324835. License: CC BY-NC-SA 3.0 IGO (2019).

[4] Cheesbrough M (2009). District Laboratory Practice in Tropical Countries Part 1.

[5] Gupta I, Guin P (2010). Communicable diseases in the South-East Asia Region of the World Health Organization: towards a more effective response. Bull. World Health Organ..

[6] Bhutta ZA, Sommerfeld J, Lassi ZS, Salam RA, Das JK (2014). Global burden, distribution, and interventions for infectious diseases of poverty. Infect. Dis. Poverty.

[7] Bendesky A, Menéndez D (2001). Metronidazol: una visión integral. Rev. Fac. Med. UNAM.

[8] Ohnishi K (2014). Subjective adverse reactions to metronidazole in patients with amebiasis. Parasitol. Int..

[9] Singh S, Shrivastav AB, Sharma RK (2009). The epidemiology of gastrointestinal parasitism and body condition in free-ranging herbivores. J. Threat. Taxa.

[10] Martínez-Gómez F, Fuentes-Castro BE, Bautista-Garfias CR (2011). The intraperitoneal inoculation of *Lactobacillus casei* in mice induces total protection against *Trichinella spiralis* infection at low challenge doses. Parasitol. Res..

[11] Newman DJ, Cragg GM (2012). Natural products as sources of new drugs over the 30 years from 1981 to 2010. J. Nat. Prod..

[12] Brian PW, Hemming HG (1947). Production of antifungal and antibacterial substances by fungi; preliminary examination of 166 strains of fungi imperfecti. Microbiol..

[13] Zhang XY, Bao J, Wang GH, He F, Xu XY, Qi SH (2012). Diversity and antimicrobial activity of culturable fungi isolated from six species of the South China Sea gorgonians. Microb. Ecol..

[14] Cowan MM (1999). Plant products as antimicrobial agents. Clin. Microbiol. Rev..

[15] Kirkup BC (2006). Bacteriocins as oral and gastrointestinal antibiotics: Theoretical considerations, applied research, and practical applications. Curr. Med. Chem..

[16] Gillor O, Ghazaryan L (2007). Recent advances in bacteriocin application as antimicrobials. Recent Pat. Antiinfect. Drug Discov..

[17] Bode HB (2009). Entomopathogenic bacteria as a source of secondary metabolites. Curr. Opin. Chem. Biol..

[18] Cai D, Zhu C, Chen S (2017). Microbial production of nattokinase: current progress, challenge and prospect. World J. Microbiol. Biotechnol..

[19] Shi YM, Bode HB (2018). Chemical language and warfare of bacterial natural products in bacteria-nematode-insect interactions. Nat. Prod. Rep..

[20] Shi YM, Hirschmann M, Shi YN, Ahmed S, Abebew D, Tobias NJ, Bode HB (2022). Global analysis of biosynthetic gene clusters reveals conserved and unique natural products in entomopathogenic nematode-symbiotic bacteria. Nat. Chem..

[21] Boemare NE, Gaugler R (2002). Biology, Taxonomy and Systematics of *Photorhabdus* and *Xenorhabdus*. Entomopathogenic Nematolog.

[22] Hazir S, Stackebrant E, Lang E, Ehlers RU, Keskin N (2004). Two new subspecies of *Photorhabdus luminescens*, isolated from *Heterorhabditis bacteriophora* (Nematoda: Heterorhabditidae): *Photorhabdus luminescens* subsp. kayaii subsp. Nov. and *Photorhabdus luminescens* subsp. *thraciaensis* subsp. nov. Syst. Appl. Microbiol..

[23] Gulcu B, Cimen H, Raja RK, Hazir S (2017). Entomopathogenic nematodes and their mutualistic bacteria: Their ecology and application as microbial control agents. Biopestic. Int..

[24] Shapiro-Ilan DI, Hazir S, Glaser I, Kogan M, Heinrichs EA (2020). Advances in Use of Entomopathogenic Nematodes in Integrated Pest Management. Integrated Management of Insect Pests: Current and Future Developments.

[25] Goodrich-Blair H, Clarke DJ (2007). Mutualism and pathogenesis in *Xenorhabdus* and *Photorhabdus*: two roads to the same destination. Mol. Microbiol..

[26] Chaston JM (2011). The entomopathogenic bacterial endosymbionts *Xenorhabdus* and *Photorhabdus*: Convergent lifestyles from divergent genomes. PLoS ONE.

[27] Akhurst RJ (1980). Morphological and functional dimorphism in *Xenorhabdus* spp. bacteria symbiotically associated with the insect pathogenic nematodes *Neoaplectana* and *Heterorhabditis*. J. Gen. Microbiol..

[28] Akhurst RJ (1982). Antibiotic activity of *Xenorhabdus* spp., bacteria symbiotically associated with insect pathogenic nematodes of the families Heterorhabditidae and Steinernematidae. J. Gen. Microbiol..

[29] Webster NS, Negri AP, Webb RI, Hill RT (2002). A spongin-boring alpha-proteobacterium is the etiological agent of disease in the Great Barrier Reef sponge *Rhopaloeides odorabile*. Mar. Ecol. Prog. Ser..

[30] Furgani G (2008). *Xenorhabdus* antibiotics: A comparative analysis and potential utility for controlling mastitis caused by bacteria. J. Appl. Microbiol..

[31] Chen J, Ding M, Pederson DS (1994). Binding of TFIID to the CYC1 TATA boxes in yeast occurs independently of upstream activating sequences. Proc. Natl. Acad. Sci. USA.

[32] Shapiro-Ilan DI, Han R, Qiu X, Morales-Ramos JA, Rojas MG, Shapiro-Ilan DI (2014). Production of Entomopathogenic Nematodes. Mass Production of Beneficial Organisms: Invertebrates and Entomopathogens.

[33] Fang X, Zhang M, Tang Q, Wang Y, Zhang X (2014). Inhibitory effect of *Xenorhabdus nematophila* TB on plant pathogens *Phytophthora capsici* and *Botrytis cinerea* in vitro and in planta. Sci. Rep..

[34] Hazir S (2016). Relative potency of culture supernatants of *Xenorhabdus* and *Photorhabdus* spp. on growth of some fungal phytopathogens. Eur. J. Plant Pathol..

[35] Cimen H (2021). Antifungal activity of different Xenorhabdus and Photorhabdus species against various fungal phytopathogens and identification of the antifungal compounds from *X. szentirmaii*. Appl. Microbiol. Biotechnol..

[36] Sergeant M (2006). Identification, typing, and insecticidal activity of *Xenorhabdus* isolates from entomopathogenic nematodes in United Kingdom soil and characterization of the xpt toxin loci. Appl. Environ. Microbiol..

[37] Mona HA, Aly NAH (2009). Insecticidal activity and genetic characterization of four bacterial isolates of *Xenorhabdus* and *Photorhabdus* associated with entomopathogenic nematodes. Pest Technol..

[38] Vitta A (2018). Larvicidal activity of *Xenorhabdus* and *Photorhabdus* bacteria against *Aedes aegypti* and *Aedes albopictus*. Asian Pac. J. Trop. Biomed..

[39] Grundmann F (2014). Antiparasitic chaiyaphumines from entomopathogenic *Xenorhabdus* sp. PB61.4. J. Nat. Prod..

[40] Antonello AM (2019). Anti-*Trypanosoma* activity of bioactive metabolites from *Photorhabdus luminescens* and *Xenorhabdus nematophila*. Exp. Parasitol..

[41] Machado RAR (2018). Whole-genome-based revisit of *Photorhabdus* phylogeny: Proposal for the elevation of most *Photorhabdus* subspecies to the species level and description of one novel species *Photorhabdus bodei* sp. nov., and one novel subspecies *Photorhabdus laumondii* subsp. *clarkei* subsp. nov. Int. J. Syst. Evol. Microbiol..

[42] Tobias NJ (2017). Natural product diversity associated with the nematode symbionts *Photorhabdus* and *Xenorhabdus*. Nat. Microbiol..

[43] Bode E (2019). Promoter activation in Δhfq mutants as an efficient tool for specialized metabolite production enabling direct bioactivity testing. Angew. Chem. Int. Ed..

[44] Dönmez-Özkan H (2019). Nematode-associated bacteria: Production of antimicrobial agent as a presumptive nominee for curing endodontic infections caused by *Enterococcus faecalis*. Front. Microbiol..

[45] Houard J (2013). Cabanillasin, a new antifungal metabolite, produced by entomopathogenic *Xenorhabdus cabanillasi* JM26. J. Antibiot..

[46] San-Blas E, Carrillo Z, Parra Y (2012). Effect of *Xenorhabdus* and *Photorhabdus* bacteria and their exudates on *Moniliophthora roreri*. Arch. Phytopathol..

[47] Ng KK, Webster JM (1997). Antimycotic activity of *Xenorhabdus bovienii* (Enterobacteriaceae) metabolites against *Phytophthora infestans* on potato plants. Can. J. Plant Pathol..

[48] Muangpat P (2017). Screening of the antimicrobial activity against drug resistant bacteria of *Photorhabdus* and *Xenorhabdus* associated with entomopathogenic nematodes from Mae Wong National Park, Thailand. Front. Microbiol..

[49] Pérez-Serrano J (2000). In vitro shock response to different stressors in free living and pathogenic *Acanthamoeba*. Int. J. Parasitol..

[50] Heredero-Bermejo I, Martin C, Soliveri J, Copa-Patiño J, Pérez-Serrano J (2012). *Acanthamoeba castellanii*: In vitro UAH-T17c3 trophozoite growth study in different culture media. Parasitol. Res..

[51] Debnath A (2013). In vitro efficacy of corifungin against *Acanthamoeba castellanii* trophozoites and cysts. Antimicrob. Agents Chemother..

[52] Latifi A, Salimi M (2020). Growth comparison of *Acanthamoeba* genotypes T3 and T4 in several culture media. Heliyon.

[53] Axelsson-Olsson D, Olofsson J, Ellström P, Waldenström J, Olsen B (2009). A simple method for long-term storage of *Acanthamoeba* species. Parasitol. Res..

[54] Clark CG, Diamond LS (2002). Methods for cultivation of luminal parasitic protists of clinical importance. Clin. Microbiol. Rev..

[55] Diamond LS, Harlow DR, Cunnick CC (1978). A new medium for the axenic cultivation of *Entamoeba histolytica* and other *Entamoeba*. Trans. R. Soc. Trop. Med. Hyg..

[56] Rangel-Castañeda IA (2018). Amoebicidal activity of curcumin on *Entamoeba histolytica* trophozoites. J. Pharm. Pharmacol..

[57] Lossick JG, Muller M, Gorrell TE (1986). In vitro drug susceptibility and doses of metronidazole required for cure in cases of refractory vaginal trichomoniasis. J. Infect. Dis..

[58] Fumarola L, Spinelli R, Brandonisio O (2004). In vitro assays for evaluation of drug activity against *Leishmania* spp. Res. Microbiol..

[59] Veiga-Santos P (2012). Effects of amiodarone and posaconazole on the growth and ultrastructure of *Trypanosoma cruzi*. Int. J. Antimicrob. Agents..

[60] Meira C (2015). In vitro and in vivo antiparasitic activity of *Physalis angulata* L. concentrated ethanolic extract against *Trypanosoma cruzi*. Phytomedicine.

[61] Baig AM, Iqbal J, Khan NA (2013). In vitro efficacies of clinically available drugs against growth and viability of an *Acanthamoeba castellanii* keratitis isolate belonging to the T4 genotype. Antimicrob. Agents Chemother..

[62] Innocente A (2014). Anti-*Trichomonas vaginalis* activity from triterpenoid derivatives. Parasitol. Res..

[63] Kuhn DM, Balkis M, Chandra J, Mukherjee PK, Ghannoum MA (2003). Uses and limitations of the XTT assay in studies of *Candida* growth and metabolism. J. Clin. Microbiol..

[64] Wainwright M (2010). Dyes, trypanosomiasis and DNA: a historical and critical review. Biotech. Histochem..

[65] Pertiwi YD (2019). Antimicrobial photodynamic therapy with the photosensitizer TONS504 eradicates *Acanthamoeba*. Photodiagn. Photodyn. Ther..

[66] Bode E (2015). Simple "on-demand" production of bioactive natural products. ChemBioChem.

[67] Wenski SL (2020). Fabclavine diversity in *Xenorhabdus* bacteria. Beilstein J. Org. Chem..

[68] SPSS v. 20.0 for Windows. SPSS Inc, Chicago, IL, USA (2011).

[69] Brachmann AO, Bode HB (2013). Identification and bioanalysis of natural products from insect symbionts and pathogens. Adv. Biochem. Eng. Biotechnol..

[70] Dreyer J, Malan A, Dicks L (2018). Bacteria of the genus *Xenorhabdus*, a novel source of bioactive compounds. Front. Microbiol..

[71] Chacón-Orozco JG (2020). Antifungal activity of the secondary metabolites and volatile compounds of *Xenorhabdus* spp. and *Photorhabdus* spp., against the soybean pathogenic *Sclerotinia scleortiorum*. Sci. Rep..

[72] Incedayi G (2021). Relative potency of a novel acaricidal compound from *Xenorhabdus*, a bacterial genus mutualistically associated with entomopathogenic nematodes. Sci. Rep..

[73] Fuchs S, Grundmann F, Kurz M, Kaiser M, Bode H (2014). Fabclavines: Bioactive peptide-polyketide-polyamino hybrids from *Xenorhabdus*. ChemBioChem.

[74] Wenski SL, Berghaus N, Keller N, Bode HB (2021). Structure and biosynthesis of deoxy-polyamine in *Xenorhabdus bovienii*. J. Ind. Microbiol. Biotechnol..

[75] Masschelein J (2013). A PKS/NRPS/FAS hybrid gene cluster from *Serratia plymuthica* RVH1 encoding the biosynthesis of three broad spectrum, Zeamine-related antibiotics. PLoS ONE.

[76] Masschelein J (2015). The zeamine antibiotics affect the integrity of bacterial membranes. Appl. Environ. Microbiol..

[77] Kim IH (2017). The insect pathogenic bacterium *Xenorhabdus innexi* has attenuated virulence in multiple insect model hosts yet encodes a potent mosquitocidal toxin. BMC Genom..

[78] Park D (2009). Genetic analysis of xenocoumacin antibiotic production in the mutualistic bacterium *Xenorhabdus nematophila*. Mol. Microbiol..

[79] Reimer D, Pos KM, Thines M, Gürün M, Bode HB (2011). A natural prodrug activation mechanism in nonribosomal peptide synthesis. Nat. Chem. Biol..

[80] Reimer D (2013). Rhabdopeptides as insect-specific virulence factors from entomopathogenic bacteria. ChemBioChem.

[81] Zhou T (2011). Global transcriptional responses of *Bacillus subtilis* to xenocoumacin 1. J. Appl. Microbiol..

[82] Challinor VL, Bode HB (2015). Bioactive natural products from novel microbial sources. Ann. N.Y. Acad. Sci..

[83] McInerney BV, Taylor WC, Lacey MJ, Akhurst RJ, Gregson RP (1991). Biologically active metabolites from *Xenorhabdus* spp. Part 2. Benzopyran-1-one derivatives with gastroprotective activity. J. Nat. Prod..

[84] McInerney BV, Taylor WC, Lacey MJ, Akhurst RJ, Gregson RP (1991). Biologically active metabolites from *Xenorhabus* spp. Part 2. Benzopyrane-1-one derivatives with gastroprotective activity. J. Nat. Prod..

[85] Li J, Chen G, Webster JM (1995). Antimicrobial metabolites from a bacteria symbiont. J. Nat. Prod..

[86] Joshi A, Verma M, Chakravorty M (1982). Thiolutin-resistant mutants of *Salmonella typhimurium*. Antimicrob. Agents Chemother..

[87] Oliva B, O'Neill A, Wilson JM, O'Hanlon PJ, Chopra I (2001). Antimicrobial properties and mode of action of the pyrrothine holomycin. Antimicrob. Agents Chemother..

[88] Gualtieri M, Aumelas A, Thaler JO (2009). Identification of a new antimicrobial lysine-rich cyclolipopeptide family from *Xenorhabdus nematophila*. J. Antibiot..

